# A Dual-Mode Flexible Sensor with Capacitive–Resistive Hybrid Response for Bolt Loosening Monitoring

**DOI:** 10.3390/s26020578

**Published:** 2026-01-15

**Authors:** Yan Ping, Kechen Li, Chao Yuan, Ding Guo, Yuanyuan Yang

**Affiliations:** 1 Dongfang Turbine Co., Ltd., Deyang 618000, China; 2 School of Aerospace Engineering, Xiamen University, Xiamen 361000, China

**Keywords:** flexible sensor, dual-mode sensing, capacitive sensing, resistive sensing, structural health monitoring, bolt loosening

## Abstract

The structural health monitoring of bolted connections is important for ensuring the safety and reliability of engineering systems, yet conventional sensing technologies struggle to balance detection range and sensitivity. This study presents a flexible sensor with a hybrid capacitive–resistive sensing mechanism, designed to overcome the limitations of single-mode sensors. By integrating a hierarchically structured composite layer with tailored material properties, the sensor achieves a seamless transition between sensing modes across different pressure ranges. It exhibits high sensitivity in both low-pressure and high-pressure regions, enabling the reliable detection of preload variations in bolted connections. Experimental validation confirms its cyclic durability and rapid response to mechanical changes, demonstrating good potential for real-time monitoring in aerospace and industrial systems.

## 1. Introduction

The integrity of bolted connections represents one of the most critical aspects in structural engineering systems, ranging from aerospace assemblies to industrial equipment [1,2,3]. These connections are subjected to various dynamic loading conditions, including vibration, thermal cycling, and mechanical stress, which can lead to gradual loosening over time. The consequences of bolt loosening can be severe, potentially resulting in catastrophic structural failures, equipment damage, and significant safety hazards [4,5]. Traditional monitoring methods, such as torque wrenches and ultrasonic testing, have inherent limitations for continuous health assessment [6,7,8,9,10,11]. These conventional approaches are typically offline, require direct physical access to the monitored components, and cannot provide real-time feedback essential for predictive maintenance strategies. The development of reliable, continuous monitoring technologies for bolted joints has therefore become an urgent research priority in structural health monitoring.

Recent advancements in flexible sensor technology have opened new possibilities for embedded structural health monitoring systems [12,13,14,15]. The unique advantages of flexible sensors, including their conformability, ease of integration, and sensitivity to mechanical stimuli, make them ideal candidates for bolt monitoring applications. Most existing flexible pressure sensors operate on a single sensing mechanism, such as resistive or capacitive [16,17,18,19,20]. Microstructural engineering is considered a primary approach to address the inherent trade-off between high sensitivity and a wide working range in pressure sensors. Through the design of specific surface microtopography, sensor performance can be significantly optimized [21,22,23,24]. However, relying solely on microstructural design still presents numerous challenges, as a performance ceiling exists that is difficult to overcome when balancing sensitivity and detection range. This fundamental trade-off has remained a significant challenge in sensor development, particularly for applications requiring monitoring across wide pressure spectra, such as bolt preload monitoring from initial assembly to full-service conditions.

In this work, we develop a flexible sensor with hybrid capacitive–resistive sensing capability based on an optimized carbon nanotube/polydimethylsiloxane (CNT/PDMS) composite structure. The key innovation of our sensor lies in its hierarchically structured composite layer featuring a strategically designed two-level slant pyramid microstructure system. This architecture enables a gradient response mechanism that activates different sensing modalities according to the applied pressure levels. The large dielectric pyramid structures ensure stable capacitive response under low pressure, while the conductive small pyramid structures facilitate resistive sensing at higher pressure ranges. The sensor’s design enables a seamless transition from capacitive response at low pressures to resistive response at high pressures. This dual-mode behavior allows the sensor to maintain high sensitivity across an extended dynamic range, effectively addressing the limitations of conventional single-mode sensors. The sensor’s capability to detect bolt loosening through electrical signal changes validates its potential for practical structural health monitoring applications. The implementation of this technology could improve the reliability and safety of bolted connections in various engineering systems, potentially transforming maintenance strategies from scheduled-based to condition-based approaches.

## 2. Results and Discussion

**Figure 1:**
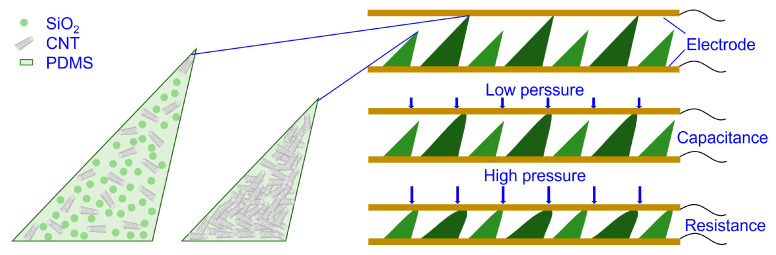
Schematic of sensor.

The developed sensor features a three-layer architecture that is specifically engineered to achieve dual-mode capacitive–resistive sensing capabilities. As illustrated in Figure 1, the core design comprises two flexible electrodes sandwiching a functional composite layer with a hierarchical microstructure. The flexible electrodes are fabricated using photolithography-patterned copper circuits on polyimide substrates, ensuring good conductivity while maintaining mechanical flexibility. The key innovation lies in the intermediate composite layer, which incorporates a strategically designed two-level slant pyramid microstructure system. This hierarchical design enables a gradient response mechanism that activates different sensing modalities according to the applied pressure levels. The composite layer utilizes polydimethylsiloxane (PDMS) as the polymer matrix, providing optimal flexibility and durability for structural monitoring applications. For the large pyramid structures, a low concentration (1 wt%) of multi-walled carbon nanotubes (CNTs) along with 0.5 wt% silica (SiO_2_) microspheres are incorporated. This specific ratio is optimized through experiments to ensure a stable capacitive response while preventing mechanical compromise from excessive filler content. Rather than using pure PDMS, the addition of CNTs enhances the dielectric properties of the composite, while the SiO_2_ microspheres act as insulating spacers that prevent the formation of conductive pathways under low-pressure conditions, thereby ensuring a pure capacitive response. Conversely, the small pyramid structures contain a higher concentration (8 wt%) of CNTs without SiO_2_ spacers, creating regions designed to form conductive networks under sufficient compression, which enables the resistive sensing mode at higher pressure ranges.

**Figure 2:**
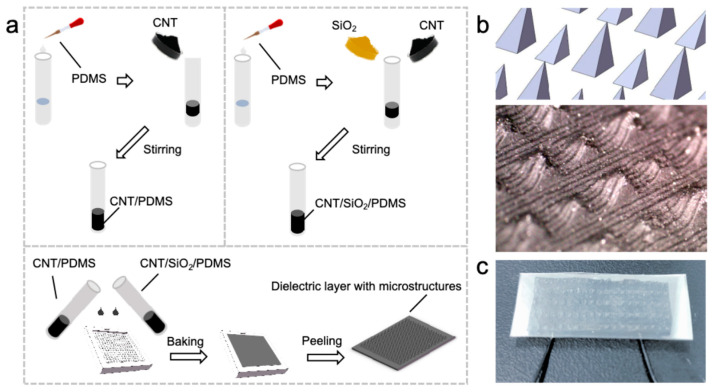
Fabrication and monography of sensor. (**a**) Fabrication process of sensor. (**b**) Microscope image of sensor. (**c**) Digital image of sensor.

The specific geometric parameters and material compositions are optimized to achieve the desired pressure-dependent modal transition. The large pyramids measure 600 μm × 600 μm at the base with a height of 800 μm, while the small pyramids feature dimensions of 500 μm × 500 μm at the base and 500 μm in height. This size differential ensures sequential engagement under increasing pressure, with the larger structures activating first for capacitive sensing and the smaller structures engaging later for resistive sensing. The fabrication process, as detailed in Figure 2a, begins with the preparation of the large pyramid through the dispersion of SiO_2_ microspheres in ethanol via ultrasonic treatment, followed by thorough mixing with the PDMS prepolymer and low-concentration CNTs. This homogeneous mixture is poured into the large pyramid cavities of a mold and cured at 80 °C for 2 h. Simultaneously, the small pyramid is prepared using a high-concentration CNT/PDMS composite without SiO_2_ spacers, cured under identical conditions. The two structurally distinct layers are then bonded by curing a new layer of PDMS above them. The microscopic characterization shown in Figure 2b confirms the integration of the hierarchical microstructure and the preservation of the distinct pyramid geometries throughout the fabrication process.

**Figure 3:**
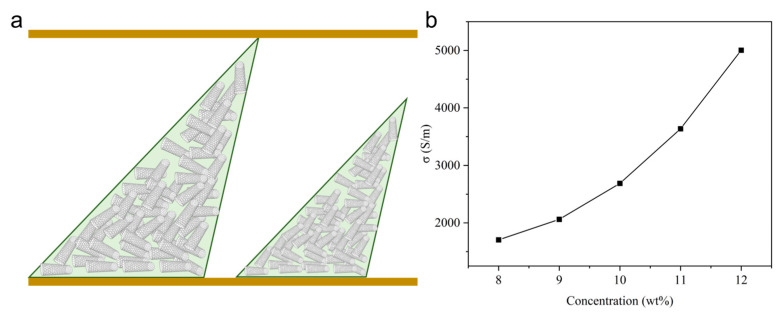
Sensor with single resistive sensing mode. (**a**) The structure of sensor with a single resistive sensing mode. (**b**) Electric conductivity according to the concentration of CNT.

**Figure 4:**
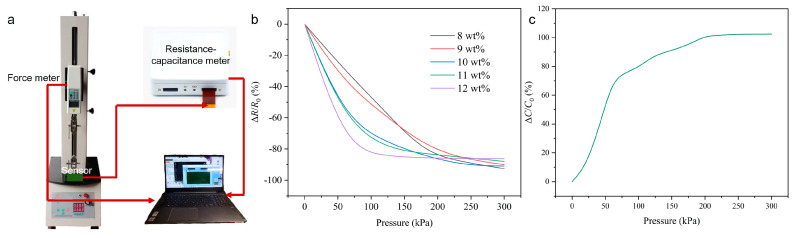
Sensing performance of sensor with single sensing mode. (**a**) Testing setup. (**b**) Electric responses of sensor working on single-resistive sensing mode. (**c**) Electric responses of sensor working on single-capacitive sensing mode.

To thoroughly investigate the advantages of the dual-mode sensing design, comparative tests on sensors operating solely in a single resistive sensing mode are conducted. During the experiments, sensors with the same two-level pyramid microstructure but using uniform CNT/PDMS composite materials are fabricated to evaluate the inherent limitations of single-mode resistive operation (Figure 3a). As the CNT concentration increased from 8 wt% to 12 wt%, the intrinsic electrical conductivity of the composite materials improved accordingly, as shown in Figure 3b. The experimental setup for the characterization tests is illustrated in Figure 4a, consisting of a force meter for applying and measuring normal pressure and a resistance–capacitance meter for simultaneous resistance and capacitance measurements. The sensor is mounted on a testing platform, with the force meter applying increasing pressure from 0 to 300 kPa while recording the corresponding electrical responses at room temperature (25 °C). The loading rate of the force meter is set as the lowest speed (2.08 kPa/s) to minimize sensor hysteresis. As shown in Figure 4b, in the low-pressure region (0–100 kPa), sensors with a higher CNT concentration (12 wt%) demonstrated higher sensitivity, exhibiting a rapid resistance decrease with applied pressure due to the formation of effective conductive pathways at lower pressure thresholds. However, this enhanced sensitivity came at the expense of the working range that the resistance change in high-CNT-concentration sensors rapidly saturated, significantly limiting their dynamic range. Conversely, sensors with a lower CNT concentration (8 wt%) showed reduced sensitivity in the low-pressure region but maintained a wider unsaturated response range in the high-pressure region. As demonstrated, optimizing sensitivity through increased filler concentration typically exacerbates response saturation. The electric response of the single capacitive mode is also tested, as shown in Figure 4c. As depicted, the capacitive-only sensor tended to saturate when the pressure exceeded 200 kPa. The results demonstrate the fundamental dilemma of single-mode resistive or capacitive operation—thereby constraining the effective working range.

**Figure 5:**
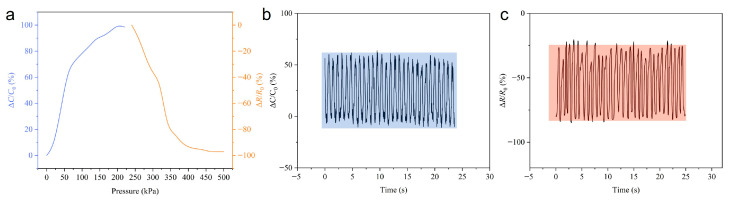
Sensing performance of sensor with dual capacitive–resistive sensing mode. (**a**) Electric response of sensor. (**b**) Stability of capacitive signal over 30 loading–unloading cycles. (**c**) Stability of resistive signal over 30 loading–unloading cycles.

Building upon the limitations of single-mode resistive sensing—where enhancing sensitivity through increased CNT concentration inevitably compromises the working range—the dual-mode sensing strategy presented in this work addresses these fundamental constraints. For the small pyramid structures, a CNT concentration of 8 wt% is chosen to achieve a broader unsaturated response range in the high-pressure region, which supports the resistive sensing mode during full-service preload monitoring. The pressure response characteristics of the dual-mode sensor, as characterized in Figure 5a, demonstrate that distinct sensing mechanisms activate across different pressure ranges to achieve comprehensive pressure monitoring coverage. In the low-pressure region (0–225 kPa), corresponding to initial bolt tightening and early-stage preload conditions, the sensor operates primarily in capacitive sensing mode. The normalized capacitance change increases rapidly, demonstrating high sensitivity (up to 0.01097 kPa^−1^) to minute pressure variations. This enhanced capacitive response at low pressures enables the precise detection of initial bolt loosening or insufficient preload during the assembly stages. The transition between operational modes occurs at approximately 225 kPa, where a well-defined handover from capacitive to resistive dominance takes place. As the capacitive response approaches saturation, the resistive response becomes progressively more pronounced, ensuring continuous monitoring capability without interruption. In the high-pressure region, which corresponds to full-service preload conditions, the resistive response mechanism dominates the sensing behavior. The normalized resistance change decreases significantly with a sensitivity up to 0.00792 kPa^−1^, providing a stable and reliable monitoring capability under high-load conditions in which conventional single-mode sensors typically exhibit saturation and performance degradation. This dual-mode operation effectively resolves the inherent conflict between sensitivity and range that plagues single-mode sensors, offering a comprehensive solution for bolt health monitoring throughout the complete service life cycle—from initial assembly through operational deployment to maintenance phases. A comparison of our sensor with reported works [25,26,27,28] demonstrates that our sensor achieves competitive sensitivity while simultaneously offering a wider detection range. The stability of the sensor is then evaluated through cyclic loading tests. As shown in Figure 5b,c, both capacitive and resistive signals exhibit good repeatability over 30 loading–unloading cycles, demonstrating the potential of the sensor design for long-term monitoring applications.

**Figure 6:**
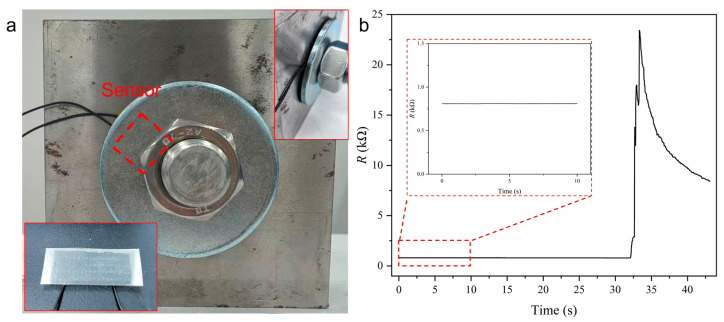
Bolt loosening monitoring tests of the sensor. (**a**) The sensor is installed between the standard washer and the workpiece surface. (**b**) Real-time monitoring data for detecting bolt loosening events.

**Figure 7:**
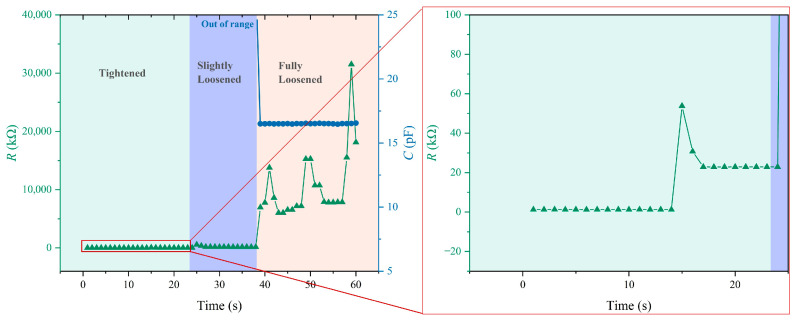
The dual-mode (resistive and capacitive) monitoring of bolt loosening. The resistive mode exhibits superior sensitivity for slight bolt loosening, whereas the capacitive mode is more suitable for identifying complete bolt loosening.

To validate the practical application of the sensor for bolt health monitoring, bolt loosening monitoring tests are carried out. The sensor is installed between the standard washer and the workpiece surface, which provides continuous preload monitoring capability, as shown in Figure 6a. During the experimental procedure, the bolt is initially tightened, establishing a preload condition that places the sensor well within its resistive sensing mode range. The real-time monitoring data, as captured in Figure 6b, reveals the sensor’s exceptional performance in detecting bolt loosening events. The resistive–capacitive dual-mode monitoring results in Figure 7 further show that the resistive mode works better for slight loosening, while the capacitive mode is suited for identifying complete bolt loosening, especially when the resistive signal approaches an open circuit. Under stable preload conditions, the resistance signal maintained good consistency, demonstrating signal stability during the initial monitoring phase. Then a loosening event is intentionally induced by applying a controlled counter-torque to the nut, reducing the preload. This preload reduction triggered an immediate and pronounced response in the resistance signal, which increases within a response time of less than 1 s. The sensor’s rapid response capability and significant signal amplitude change provide a clear detection of bolt loosening. This experimental validation demonstrates that the dual-mode sensor not only provides theoretical advantages but also delivers practical monitoring performance for bolt integrity assessment.

## 3. Conclusions

This research demonstrates a dual-mode sensing strategy that effectively bridges the gap between high sensitivity and a wide detection range in structural health monitoring. The sensor’s architecture, combining gradient microstructures with a functional material design, enables mode switching adapted to different pressure conditions. The experimental results verify its capability to maintain a stable performance under cyclic loading and accurately detect bolt preload loss in real time. The proposed technology not only addresses the fundamental limitations of existing sensors but also provides a feasible path toward embedded monitoring systems for critical connections. Although the current thickness of the sensor requires consideration during installation, ongoing design optimization is expected to further enhance its practical integration. In conclusion, this study lays a foundation for next-generation monitoring solutions that enhance safety and operational efficiency across engineering fields.

## Data Availability

The data are available on request from the authors.
